# Supplementary material for: Development, Validation and Application of a Bridging ELISA for Detection of Antibodies against GQ1001 in Cynomolgus Monkey Serum
Source: Molecules. 2023 Feb 10;28(4):1684. doi: 10.3390/molecules28041684 (PMC9964351; doi:10.3390/molecules28041684)
Supplement: Supplementary file 1 [file molecules-28-01684-s001.zip › molecules-2166063-supplementary.pdf]

Table S1: Supplementary data for Figure 1

No.	SB	IR%
1	0.998	16.4
2	1.435	44.9
3	2.313	54.2
4	1.362	32.3
5	1.109	17.1
6	1.156	26.9
7	0.998	18.7
8	1.118	13.6
9	1.024	21.7
10	1.015	20.4
11	0.967	15.4
12	0.949	17.1
13	1.091	24.6
14	1.085	16.9
15	1.087	19.4
16	1.312	15.3
17	1.709	19.4
18	1.281	28.4
19	0.968	14.3
20	1.011	13.2
21	1.034	18.5
22	0.963	13.1
23	0.991	10.2
24	0.998	10.9
25	1.267	12.8
26	1.040	17.6
27	0.979	6.5
28	1.102	22.8
29	0.977	13.7
30	1.364	7.4
31	0.994	11.9
32	1.073	13.4
33	1.609	19.2
34	1.036	16.9
35	1.025	14.3
36	1.625	9.9
37	1.066	15.2
38	1.493	14.0
39	2.402	7.4
40	1.808	6.0

41	0.968	13.2
42	0.977	14.4
43	1.023	13.6
44	2.317	56.0
45	0.967	14.0
46	1.758	9.5
47	1.065	20.4
48	1.134	13.3
49	1.057	23.0
50	1.006	13.1
51	1.406	38.1
52	0.893	19.3
53	1.058	28.5
54	1.937	53.5
55	1.266	24.7
56	0.922	15.0
57	1.379	NA
58	0.995	32.5
59	1.161	-2.2
60	0.913	18.1
61	0.816	-48.5
62	0.864	4.3
63	0.965	19.0
64	0.974	17.0
65	0.953	21.8
66	0.927	16.5
67	1.154	16.1
68	1.328	16.5
69	1.425	50.8
70	1.097	45.3
71	1.057	39.0
72	1.197	45.8
73	1.063	38.8
74	1.086	41.4
75	1.128	44.7
76	1.209	30.2
77	1.247	48.8
78	1.108	46.1
79	1.133	45.7
80	1.151	44.3
81	1.449	36.7
82	1.183	48.0
83	1.154	45.0

84	1.537	36.5
85	1.003	35.7
86	0.939	23.0
87	1.472	12.3
88	1.057	23.6
89	1.406	19.8
90	2.232	11.9
91	1.669	13.0
92	0.940	23.6
93	0.966	29.5
94	0.970	28.2
95	1.475	44.1
96	0.898	22.8
97	1.671	16.3
98	0.964	27.4
99	1.064	22.5
100	0.966	29.6
101	0.979	25.8
102	1.161	36.8
103	0.876	17.1
104	1.039	27.5
105	2.215	58.6
106	1.392	36.0
107	0.923	14.7
108	1.070	33.0
109	0.990	26.6
110	1.108	24.6
111	0.927	22.1
112	0.891	21.8
113	0.919	18.2
114	0.961	20.2
115	0.937	22.6
116	0.940	21.3
117	0.963	15.8
118	1.156	18.0
119	1.193	11.7
120	1.102	25.6
121	0.925	12.8
122	0.905	7.0
123	0.970	14.0
124	0.910	14.2
125	0.879	12.3
126	0.907	10.1

127	1.072	20.2
128	0.878	13.6
129	0.850	6.1
130	0.902	11.9
131	0.879	13.7
132	1.037	10.6
133	0.877	12.0
134	1.024	23.3
135	1.195	14.3
136	0.905	19.7
137	0.913	11.7
138	1.314	6.6
139	1.080	11.9
140	1.367	8.9
141	1.686	6.4
142	1.475	12.0
143	1.021	12.5
144	1.107	15.9
145	0.960	12.9
146	1.357	30.8
147	1.020	15.0
148	1.428	10.8
149	0.865	6.4
150	1.009	10.0
151	1.059	17.8
152	1.044	11.4
153	1.113	27.5
154	0.929	-0.2
155	1.009	-0.9
156	2.265	48.7
157	1.302	12.6
158	1.012	-2.2
159	0.995	2.9
160	0.990	-2.2
161	1.052	-5.7
162	0.978	-2.9
163	0.956	-4.0
164	0.935	-7.0
165	0.924	-6.6
166	0.986	0.8
167	0.999	-0.5
168	1.037	-5.8
169	1.175	-3.7

170	1.240	-1.1
171	1.156	9.4
172	0.920	-6.3
173	1.015	-1.6
174	1.015	-4.6
175	0.945	-4.5
176	0.925	-5.1
177	0.920	-8.9
178	1.140	-4.5
179	0.955	-7.7
180	0.931	-6.8
181	0.926	-11.5
182	0.940	-5.1
183	1.202	2.0
184	0.916	-7.8
185	0.996	-1.5
186	1.351	4.2
187	1.046	3.2
188	1.030	5.9
189	1.563	1.2
190	1.130	5.3
191	1.449	4.9
192	1.909	2.4
193	1.662	0.8
194	1.014	-1.1
195	1.008	1.5
196	1.023	4.5
197	1.275	17.3
198	0.993	-1.2
199	1.540	-1.4
200	0.987	3.9
201	1.044	0.3
202	1.054	7.7
203	1.002	1.4
204	1.110	15.2
205	1.047	13.1
206	1.062	12.3
207	2.304	51.4
208	1.369	23.9
209	1.051	5.2
210	1.070	15.0
211	0.995	10.3
212	1.102	5.3

213	0.951	9.9
214	0.957	10.8
215	0.963	9.0
216	0.918	2.7
217	0.934	6.6
218	0.942	5.6
219	0.967	5.4
220	1.129	4.9
221	1.254	11.2
222	1.083	12.5
223	0.945	2.2
224	0.964	-4.0
225	1.052	3.9
226	0.918	-2.4
227	1.004	-2.7
228	0.959	-5.8
229	1.209	6.3
230	0.999	5.8
231	0.987	-1.7
232	1.085	-1.2
233	1.045	-6.4
234	1.122	-3.0
235	0.920	-1.4
236	1.004	-3.2
237	1.346	3.3
238	0.885	8.6
239	0.975	11.4
240	1.576	2.4
241	1.178	16.0
242	1.487	8.4
243	2.112	7.8
244	1.631	4.3
245	0.978	3.1
246	1.065	12.7
247	1.011	11.4
248	1.486	28.7
249	0.959	5.3
250	1.677	3.3
251	0.923	8.8
252	1.075	11.1
253	1.016	14.4
254	1.011	10.7
255	1.253	28.5

256	0.956	7.7
257	1.140	17.7
258	2.433	52.3
259	1.374	26.3
260	0.992	1.4
261	1.090	20.8
262	1.028	9.5
263	1.126	5.0
264	0.979	4.7
265	0.928	6.8
266	0.980	6.0
267	0.929	0.8
268	1.041	9.0
269	1.042	8.5
270	1.076	10.6
271	1.223	6.2
272	1.486	5.4
273	1.183	16.2
274	0.894	-2.3
275	0.947	-2.8
276	1.004	1.3
277	1.022	10.1
278	0.926	6.1
279	0.944	7.1
280	1.163	5.5
281	1.013	7.6
282	0.941	6.2
283	0.957	3.7
284	0.924	1.6
285	1.349	6.9
286	1.003	7.8
287	1.047	9.4
288	1.390	9.9
289	0.991	1.6
290	1.004	2.3
291	1.614	4.0
292	1.127	6.5
293	1.582	6.5
294	2.273	1.2
295	1.763	4.3
296	0.977	-3.5
297	1.076	12.0
298	1.046	2.1

299	1.481	22.3
300	1.020	2.1
301	1.626	-3.0
302	0.974	5.9
303	1.098	1.7
304	1.074	12.3
305	1.032	3.3
306	1.213	20.9

Note: NA: Not Applicable.
